# Use of and barriers to adopting standardized social risk screening tools in federally qualified health centers during the first year of the COVID‐19 pandemic

**DOI:** 10.1111/1475-6773.14232

**Published:** 2023-09-16

**Authors:** Nicole C. Giron, Megan B. Cole, Kevin H. Nguyen

**Affiliations:** ^1^ Department of Health Services, Policy & Practice Brown University School of Public Health Providence Rhode Island USA; ^2^ Department of Health Law, Policy, and Management Boston University School of Public Health Boston Massachusetts USA

**Keywords:** cross‐sector collaboration, federally qualified health centers, safety net, social determinants of health, social risk factor screening

## Abstract

**Objective:**

To describe the national rate of social risk factor screening adoption among federally qualified health centers (FQHCs), examine organizational factors associated with social risk screening adoption, and identify barriers to utilizing a standardized screening tool in 2020.

**Data Source:**

2020 Uniform Data System, a 100% sample of all US FQHCs (*N* = 1375).

**Study Design:**

We used multivariable linear probability models to assess the association between social risk screening adoption and key FQHC characteristics. We used descriptive statistics to describe variations in screening tool types and barriers to utilizing standardized tools. We thematically categorized open‐ended responses about tools and barriers.

**Data Collection:**

None.

**Principal Findings:**

In 2020, 68.9% of FQHCs screened patients for any social risk factors. Characteristics associated with a greater likelihood of screening adoption included having high proportions of patients best served in a language other than English (18.8 percentage point [PP] increase, 95% CI: 6.0, 31.6) and being larger in size (10.3 PP increase, 95% CI: 0.7, 20.0). Having higher proportions of uninsured patients (14.2 PP decrease, 95% CI: −25.5, −0.3) and participating in Medicaid‐managed care contracts (7.3 PP decrease, 95% CI: −14.2, −0.3) were associated with lower screening likelihood. Among screening FQHCs, the Protocol for Responding to and Assessing Patients' Assets, Risks, and Experiences (PRAPARE) was the most common tool (47.1%). Among non‐screening FQHCs, common barriers to using a standardized tool included lack of staff training to discuss social issues (25.2%), inability to include screening in patient intake (21.7%), and lack of funding for addressing social needs (19.2%).

**Conclusions:**

Though most FQHCs screened for social risk factors in 2020, various barriers have prevented nearly 1 in 3 FQHCs from adopting a screening tool. Policies that provide FQHCs with resources to support training and workflow changes may increase screening uptake and facilitate engagement with other sectors.

**What is known on this topic**

- The COVID‐19 pandemic elucidated the importance of cross‐sector collaborations between health care, public health, and social services to jointly advance health equity, which may be facilitated through social risk screening.
- Federally qualified health centers (FQHCs) are safety‐net providers that serve over 30 million low‐income people in the United States, and many FQHCs collect social risk data.
- FQHCs are pivotal health care partners because of their social risk screening efforts, but there is limited data about the characteristics and barriers associated with social risk screening at FQHCs.

**What this study adds**

- Using a 100% sample of US FQHCs, we identified the patient‐ and organization‐level characteristics associated with social risk screening at FQHCs in 2020, the first year of the COVID‐19 pandemic.
- Despite high rates of social risk screening at FQHCs, one in three FQHCs did not screen; FQHCs with higher proportions of uninsured patients and FQHCs smaller in size were less likely to screen.
- In the first‐known national examination of barriers to standardized social risk screening at FQHCs, common barriers included lack of staff training, inability to include screening in workflow, and limited funding.

## 1 INTRODUCTION

The onset of the Coronavirus 2019 (COVID‐19) pandemic disrupted communities globally. In the United States (US), the impact of the pandemic was especially devastating for marginalized communities because it exacerbated the longstanding structural and social inequities affecting them.^1^, ^2^ Prior research has documented an increase in the prevalence of social risk factors (e.g., food insecurity, housing instability, and economic insecurity) among marginalized communities, which continues to persist years after the pandemic's onset.^3^, ^4^, ^5^ The pandemic's devastating impact has intensified national attention on the social determinants of health as drivers of equity. Some researchers and advocates suggest that cross‐sector efforts—which can include developing connections across organizations and sharing data, like data on social risk factors—can assist in addressing inequities.^6^

Federally qualified health centers (FQHCs) are essential partners for engaging in cross‐sector efforts that connect health care with social services and public health. The nearly 1400 FQHCs throughout the US serve as an essential safety‐net system for low‐income individuals and their families. FQHCs serve over 30 million people throughout the country and are the primary care provider for one in three people living in poverty and one in five uninsured individuals.^7^, ^8^ Aligning with FQHCs can strengthen the impact of cross‐sector collaborations because FQHCs can be key partners in reaching community members, sharing data (such as social risk data), and advocating for policy change.^9^, ^10^ Some FQHCs have experience collaborating with diverse partners in culturally‐grounded cross‐sector efforts to improve health outcomes among minoritized racial and ethnic communities.^11^, ^12^ A recent study of national FQHC data found that 71% of FQHCs reported collecting social risk data from their patients in 2019—information that can be integral to informing and reinforcing the impact of cross‐sector efforts.^13^ For example, FQHCs have participated in cross‐sector efforts by engaging in data collection and referral mechanisms to help address housing instability and food insecurity.^14^, ^15^ Others have used social risk data to build relationships with local agencies and develop interventions that provide additional food, transportation, and housing benefits to their patients.^16^, ^17^ However, to successfully engage FQHCs in cross‐sector collaboratives, we must understand the current social risk screening environment and related barriers.

Currently, the use of standardized screening tools is being emphasized to promote consistent data collection. Standardized data can be leveraged for efforts that rely on data sharing, like cross‐sector collaboratives. However, the implementation of social risk screening tools in the patient workflow can be resource‐intensive, and standardized tools may not meet the FQHC workforce or patient population needs. Additionally, a defining feature of FQHCs is their use of a Prospective Payment System (PPS) payment methodology, in which FQHCs are reimbursed an enhanced, predetermined, comprehensive per‐visit rate that is meant to provide FQHCs with financial consistency and predictability. While FQHCs increasingly participate in alternative payment models, those that are reimbursed via PPS may have limited financial flexibility to provide services that are not otherwise built into the PPS system, such as screening for and addressing social risk factors.^18^, ^19^ Exploring the specific barriers that FQHCs report to utilizing standardized tools, particularly among those that are not screening at all, may inform policy changes that can better support FQHC engagement in social risk screening and cross‐sector collaboratives.

This study builds on prior work^13^ by utilizing more recent standardized FQHC data to report on the state of social risk screening among FQHCs during the first year of the COVID‐19 pandemic and by exploring barriers to standardized screening tool adoption. The specific objectives of this study were to (1) describe the national rate of social risk screening adoption and use of standardized screening tools among FQHCs in 2020, (2) to examine organizational factors associated with screening adoption, and (3) identify barriers to utilizing a standardized screening tool among non‐screening FQHCs. We also examined characteristics of non‐screening FQHCs based on their reported barriers to utilizing standardized screening tools.

## 2 METHODS

### 2.1 Data source and study sample

This study used the 2020 Health Resources and Services Administration's (HRSA) Uniform Data System (UDS). The UDS is a standardized dataset that is collected annually and captures organization‐level information on the patient characteristics, performance, and operations of FQHCs during a full calendar year.^20^ All FQHCs that are funded through the HRSA Health Center Program are required to submit their UDS annually following the end of the calendar year of interest, where each year of data reflects services rendered and FQHC capabilities as of December 31st of that calendar year. Our study sample, which includes 1375 FQHCs, represents a 100% sample of all FQHCs in the 50 US states, the District of Columbia, and eight US territories, which together served 28.6 million patients in 2020.^7^

### 2.2 Measures

The primary outcome was whether an FQHC screened for any social risk factors in 2020. The UDS asked, “*Does your health center collect data on individual patients' social risk factors*, *outside of the data countable in the UDS?”* FQHCs could indicate one of three options: Yes; No, but we are in the planning stages to collect this information; or No, and we are not planning to collect this information. Responses were collapsed into a binary outcome (yes/no). We provide the distribution of health center characteristics disaggregated by the three original options in Table S1.

FQHCs who reported screening were asked to select which standardized tools they used and could select multiple screeners. The possible responses included: Accountable Health Communities Screening Tools; Upstream Risks Screening Tool and Guide; iHELLP; Recommend Social and Behavioral Domains for EHRs; Protocol for Responding to and Assessing Patients' Assets, Risks, and Experiences (PRAPARE); Well Child Care, Evaluation, Community Resources, Advocacy, Referral, Education (WE CARE); WellRx; Health Leads Screening Toolkit; Other (please describe); and We do not use a standardized screener.

FQHCs who did not report screening for social risks utilizing a standardized tool were prompted to indicate their reasons for not utilizing a standardized tool and were able to select among the following options: Have not considered/unfamiliar with assessments; Lack of funding for addressing these unmet social needs of social patients; Lack of training for staff to discuss these issues with patients; Inability to include with patient intake and clinical workflow; Not needed; Other (please describe). FQHCs could select multiple barriers.

Of note, questions about standardized social risk screening practices at FQHCs were incorporated for the first time into the 2019 UDS report.^21^ These questions focused on whether or not FQHCs were screening their patients for social risk factors and, if so, which tools they were using. In 2020, a question assessing barriers to social risk screening using a standardized tool was added to the UDS report for the first time.^22^

### 2.3 Study design and analysis

We first calculated rates of social risk screening adoption among FQHCs in 2020 and compared characteristics of FQHCs by social risk screening status (i.e., screening vs. not screening) using Wilcoxon‐Mann–Whitney tests. Building upon previous work, characteristics included FQHC‐level patient composition (e.g., age, gender, race and ethnicity, health insurance coverage type, sexual orientation, and gender identity [SOGI] minority status, homelessness status, veteran status, and English proficiency), HRSA Health Center Program grant type, rural versus urban setting, FQHC size (number of unique patients), overall FQHC revenue (measured as the total of non‐patient‐related revenue, such as grants and contracts), revenue per patient, electronic health record (EHR) use, participation in a Medicaid‐managed care contract, and state Medicaid expansion status as of January 2020.^23^, ^24^ We also explored screening differences by the proportion of patients with select chronic condition diagnoses (i.e., diabetes mellitus diagnosis and hypertension diagnosis) and select behavioral health diagnoses (i.e., depression diagnosis, anxiety disorder diagnosis, alcohol‐related disorders, and other substance‐related disorders).

We then assessed the association between social risk screening adoption and key FQHC characteristics using multivariable linear probability models. We adjusted for covariates that had a statistically significant difference between groups before adjustment or had been identified by prior literature to have a relationship with social risk screening. These covariates, all measured in quartiles, included the percentage of patients who were: pediatric; older adults; American Indian/Alaska Native, non‐Hispanic; Asian, non‐Hispanic; Black, non‐Hispanic; Hispanic; insured by Medicaid; insured by Medicare; uninsured; sexual or gender minorities; experiencing homelessness; and best served in a language other than English. We also included covariates (measured in quartiles) for the percentage of patients diagnosed with: diabetes mellitus; hypertension; anxiety disorders; depression; alcohol‐related disorders; and substance‐related disorders. FQHC‐level covariates included size (measured in quartiles); geographic area; revenue per 1000 patients served; participation in a Medicaid‐managed care contract; and presence in a Medicaid expansion state. We present both unadjusted and adjusted associations between FQHC‐level characteristics and screening for social risk factors. Additionally, we omitted FQHCs located in the US territories from the main analysis (*n* = 33), but we reran the main model to include these FQHCs in sensitivity analysis.

Next, we examined the types of standardized screening tools used, if any, among FQHCs that collected social risk data. We also examined reported barriers to screening using a standardized tool among FQHCs who reported not screening for social risk factors. In supplemental analyses, we explored barriers among FQHCs who reported screening with a non‐standardized tool, as well. Because FQHCs had the option of selecting “Other” for the questions on used tools and barriers to screening, open‐ended responses were assessed and either grouped with existing survey responses or, when applicable, added to a new category. Lastly, we explored characteristics of non‐screening FQHCs by top reasons for not collecting social risk data using a standardized tool.

Analyses were performed using Stata, version 17.0, between 2022 and 2023. This study was exempt from the institutional review board (IRB).

## 3 RESULTS

### 3.1 Characteristics associated with screening implementation

Over two‐thirds of all FQHCs reported screening for social risk factors in 2020 (68.9%) (Table 1). While FQHCs that screened versus did not screen for social risk factors were similar in most characteristics, FQHCs that did not screen reported serving a higher proportion of patients who were American Indian or Alaska Native (3.4% vs. 1.3%, *p* < 0.001) or uninsured (26.6% vs. 22.4%, *p* < 0.001) compared to FQHCs that screened. Additionally, FQHCs that did not screen had lower proportions of patients who were enrolled in Medicaid (39.2% vs. 43.2%, *p* < 0.001) or had a diagnosis of anxiety disorder (9.9% vs. 11.2%, *p* < 0.001) or depression (9.7% vs. 10.9%, *p* = 0.003); were more concentrated in rural areas (47.2% vs. 39.7%, *p* = 0.009); had fewer unique patients (16,102 patients vs. 22,914 patients, *p* < 0.001); reported less grant revenue ($7.6 million vs. $9.6 million); and were less concentrated in states that expanded Medicaid (65.7% vs. 74.0%, *p* = 0.002) compared to FQHCs that screened for social factors. There were similar patterns between groups when disaggregated by the original response options (Table S1), as well as when we considered the unadjusted associations between screening and FQHC characteristics using quartile distributions (Table S2).

**TABLE 1:** Federally qualified health center characteristics by social risk screening adoption status (2020).

Characteristic	Do not screen *N* = 428 (31.1%)	Currently screen *N* = 947 (68.9%)	*p*‐Value of difference
Age, %			
Pediatric (0–17 years)	21.2 (13.9)	22.6 (12.6)	0.058*
Adult (18–64 years)	64.1 (11.8)	64.1 (11.2)	0.929
Older adult (65+ years)	11.6 (7.2)	11.2 (6.5)	0.266
Gender, %			
Female	56.5 (6.5)	56.6 (6.0)	0.790
Race/Ethnicity, %			
American Indian or Alaska Native, non‐Hispanic	3.4 (13.2)	1.3 (6.1)	<0.001***
Asian, non‐Hispanic	2.7 (8.1)	3.3 (9.4)	0.216
Black, non‐Hispanic	19.3 (23.9)	17.8 (21.9)	0.251
Hispanic	25.6 (27.2)	27.9 (26.8)	0.148
Other, non‐Hispanic	1.2 (1.7)	1.3 (2.4)	0.137
White, non‐Hispanic	39.9 (29.9)	41.5 (30.1)	0.377
Insurance coverage, %			
Medicaid	39.2 (19.9)	43.2 (17.3)	<0.001***
Medicare	11.7 (8.0)	11.8 (7.2)	0.820
Other public (e.g., state government programs)	0.7 (2.7)	0.7 (2.1)	0.983
Private	21.7 (13.5)	21.9 (12.8)	0.808
Uninsured	26.6 (19.9)	22.4 (16.3)	<0.001***
Other patient characteristics, %			
Sexual orientation/gender identity minority	7.9 (12.4)	8.9 (13.5)	0.184
Experiencing homelessness	6.8 (18.1)	6.8 (16.6)	0.969
Best served in non‐english language	18.1 (23.2)	20.3 (22.4)	0.105
Veteran	1.7 (1.9)	1.8 (2.3)	0.646
Diabetes diagnosis	10.1 (4.8)	9.8 (3.9)	0.213
Hypertension diagnosis	20.2 (9.8)	19.3 (8.3)	0.074*
Anxiety disorder diagnosis	9.9 (6.4)	11.2 (6.6)	<0.001***
Depression diagnosis	9.7 (6.9)	10.9 (7.1)	0.003***
Alcohol‐related diagnosis	1.8 (4.2)	1.8 (2.4)	0.719
Other substance‐related disorder diagnosis	2.7 (5.6)	3.1 (4.6)	0.149
FQHC‐level characteristics			
Section 330(e) Community Health Center Grant Funding, %	94.9 (22.1)	94.8 (22.2)	0.979
Section 330(h) Health Care for the Homeless Grant Funding, %	19.6 (39.8)	22.7 (41.9)	0.200
Section 330(g) Migrant Health Center Grant Funding, %	12.9 (33.5)	12.7 (33.3)	0.927
Section 330(i) Public Housing Primary Care grant funding, %	7.0 (25.6)	8.1 (27.4)	0.472
Rural, %	47.2 (50.0)	39.7 (49.0)	0.009***
Unique patient count, *n*	16,102 (19,102)	22,914 (28,873)	<0.001***
Total non‐patient revenue, $	7,601,722 (9,884,998)	9,579,401 (15,271,836)	0.014**
Revenue per patient, $	745 (930)	681 (1873)	0.500
EHR Use, %	99.3 (8.4)	99.7 (5.6)	0.317
Medicaid‐managed care contract, %	23.8 (42.7)	24.2 (42.8)	0.888
State‐level characteristics, %			
Medicaid expansion as of 2020	65.7 (47.5)	74.0 (43.9)	0.002***

*Note*: This table reports proportional means and standard deviations except where otherwise noted. Wilcoxon‐Mann–Whitney tests were used to assess statistical differences. EHR is an electronic health record. Total Non‐Patient Revenue includes income‐related non‐patient receipts (e.g., grants and contracts). Medicaid expansion percentages exclude federally qualified health centers located in United States territories (*N* = 1342).

* *p* < 0.10.

** *p* < 0.05.

*** *p* < 0.01.

In adjusted models, characteristics that were associated with a greater likelihood of screening included having a higher proportion of patients best served in a language other than English (adjusted difference between FQHCs with highest vs. lowest proportion = 18.8 percentage points [PP] increase, 95% CI = 6.0, 31.6) and largest (vs. smallest) number of unique patients (adjusted difference = 10.3 PP increase, 95% CI = 0.7, 20.0). Characteristics associated with a lower likelihood of screening included having a higher (vs. lower) concentration of medically uninsured patients (adjusted difference = 14.2 PP decrease, 95% CI = −25.5, −0.3) and participating in a Medicaid‐managed care contract (adjusted difference = 7.3 PP decrease, 95% CI = −14.2, −0.3).

In sensitivity analyses, when FQHCs located in US territories were included in the model, results were similar (Table S3).

### 3.2 Screening tool types

**FIGURE 1:**
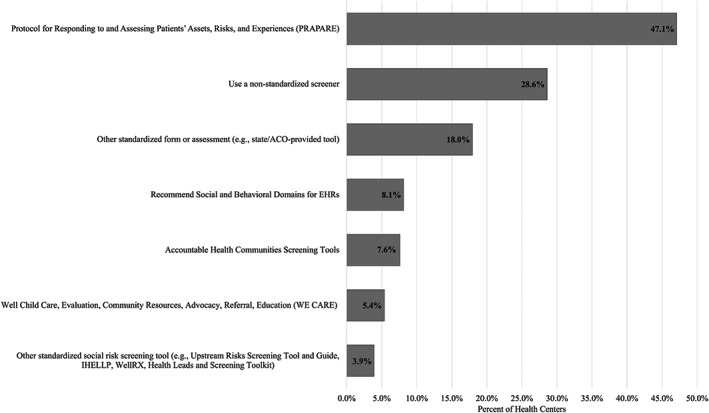
Use of screening tools among federally qualified health centers collecting social risk data (*N* = 947). Percentages do not add to 100% because federally qualified health centers were able to select multiple response options. ACO is an Accountable Care Organization. EHR is an electronic health record.

Almost half (47.1%) of the FQHCs that collected social risk data in 2020 indicated that they were using the PRAPARE tool (Figure 1). FQHCs could report using more than one standardized screener. Among FQHCs that collected social risk data, 43.0% used a standardized tool other than PRAPARE, including 18.0% that reported using a standardized tool that was not listed in the survey instrument (i.e., selecting “other”), such as tools provided by an Accountable Care Organization or that were adapted from existing standardized tools such as PRAPARE. Nearly one‐third (28.6%) of FQHCs collecting social risk data indicated that they were not using a standardized screener (Table 2).

**TABLE 2:** Adjusted differences in the probability of social risk screening by federally qualified health center characteristics.

Variables	Percentage point difference (95% CI)	*p*‐value
Percent patients who are pediatric (0–17 years), %		
q1: 0% to <13.7%	Ref	Ref
q2: 13.7% to <22.4%	2.2 (−5.6, 9.9)	0.587
q3: 22.4% to <30.6%	−1.3 (−10.0, 7.3)	0.763
q4: 30.6% to ≤54.5%	1.4 (−8.5, 11.4)	0.780
Percent patients who are older adults (65+ years), %		
q1: 0% to 6.7%	Ref	Ref
q2: 6.7% to <9.5%	2.8 (−5.2, 10.8)	0.486
q3: 9.5% to <14.5%	−5.6 (−15.0, 3.9)	0.248
q4: 14.5% to ≤32.6%	−0.7 (−13.2, 11.9)	0.919
Percent patients who are American Indian/Alaska Native, non‐Hispanic, %		
q1: 0%	Ref	Ref
q2: >0% to <0.2%	7.7 (−1.1, 16.4)	0.086*
q3: 0.2% to <0.6%	3.7 (−4.0, 11.5)	0.343
q4: 0.6% to ≤52.6%	1.1 (−6.8, 9.1)	0.782
Percent patients who are Asian, non‐Hispanic, %		
q1: 0% to <0.3%	Ref	Ref
q2: 0.3% to <0.8%	4.3 (−3.4, 12.0)	0.268
q3: 0.8% to <2.2%	0.6 (−9.2, 8.1)	0.901
q4: 2.2% to ≤55.4%	0.7 (−8.6, 10.0)	0.884
Percent patients who are Black, non‐Hispanic, %		
q1: 0% to <1.6%	Ref	Ref
q2: 1.6% to <8.3%	5.5 (−2.4, 13.5)	0.171
q3: 8.3% to <30.1%	2.1 (−6.9, 11.1)	0.647
q4: 30.1% to ≤88.0%	4.5 (−5.4, 14.4)	0.373
Percent patients who are Hispanic, %		
q1: 0% to <4.7%	Ref	Ref
q2: 4.7% to <16.3%	−4.1 (−13.4, 5.3)	0.397
q3: 16.3% to <42.4%	−2.6 (−14.0, 8.8)	0.653
q4: 42.4% to ≤91.7%	−0.7 (−13.6, 12.2)	0.916
Percent patients insured by Medicaid, %		
q1: 0% to <27.4%	Ref	Ref
q2: 27.4% to <41.5%	2.5 (−5.4, 10.4)	0.537
q3: 41.5% to <56.0%	2.4 (−7.3, 12.1)	0.627
q4: 56.0% to ≤79.6%	−2.8 (−14.5, 8.9)	0.642
Percent patients insured by Medicare, %		
q1: 0% to <6.3%	Ref	Ref
q2: 6.3% to <10.2%	4.3 (−4.0, 11.8)	0.294
q3: 10.2% to <16.4%	7.9 (−4.5, 15.4)	0.128
q4: 16.4% to ≤33.4%	2.1 (−14.0, 11.4)	0.753
Percent patients who are uninsured, %		
q1: 0% to <11.1%	Ref	Ref
q2: 11.1% to <19.4%	−4.7 (−12.2, 2.7)	0.211
q3: 19.4% to <31.5%	−7.7 (−16.2, 0.8)	0.075*
q4: 31.5% to ≤81.4%	−14.2 (−25.5, −0.3)	0.013**
Percent patients who identified as SOGI minority, %		
q1: 0% to <1.7%	Ref	Ref
q2: 1.7% to <3.7%	6.3 (−1.1, 13.6)	0.093
q3: 3.7% to <10.0%	3.6 (−4.0, 11.2)	0.352
q4: 10.0% to ≤68.5%	5.8 (−1.6, 13.2)	0.127
Percent patients who are experiencing homelessness, %		
q1: 0% to <0.3%	Ref	Ref
q2: 0.3% to <1.4%	4.8 (−2.5, 12.1)	0.200
q3: 1.4% to <5.0%	5.6 (−2.1, 13.2)	0.152
q4: 5.0% to ≤100%	5.0 (−3.1, 13.1)	0.228
Percent patients best served in a language other than english, %		
q1: 0% to <2.1%	Ref	Ref
q2: 2.1% to <10.4%	6.4 (−3.0, 15.7)	0.181
q3: 10.4% to <29.5%	5.1 (−6.5, 16.6)	0.389
q4: 29.5% to ≤79.8%	18.8 (6.0, 31.6)	0.004***
Percent of patients with a diabetes diagnosis, %		
q1: 0% to <7.2%	Ref	Ref
q2: 7.2% to <9.4%	−0.9 (−8.8, 6.9)	0.814
q3: 9.4% to <12.1%	4.1 (−5.3, 13.6)	0.392
q4: 12.1% to ≤22.9%	3.0 (−8.2, 14.1)	0.602
Percent patients with a hypertension diagnosis, %		
q1: 0% to <13.3%	Ref	Ref
q2: 13.3% to <18.5%	−2.0 (−10.0, 6.1)	0.631
q3: 18.5% to <24.4%	−3.4 (−13.4, 6.5)	0.500
q4: 24.4% to ≤45.0%	−6.6 (19.3, 6.1)	0.309
Percent of patients with an anxiety disorder diagnosis, %		
q1: 0% to <6.5%	Ref	Ref
q2: 6.5% to <9.8%	4.1 (−4.5, 12.6)	0.356
q3: 9.8% to <13.8%	2.4 (−8.2, 13.0)	0.660
q4: 13.8% to ≤35.4%	7.4 (−5.3, 20.1)	0.254
Percent of patients with a depression diagnosis, %		
q1: 0% to <6.1%	Ref	Ref
q2: 6.1% to <9.2%	−2.2 (−11.2, 6.8)	0.631
q3: 9.2% to <13.1%	−1.9 (−13.1, 9.4)	0.742
q4: 13.1% to ≤42.8%	−1.7 (−15.2, 11.9)	0.811
Percent of patients with an alcohol‐related disorder diagnosis, %		
q1: 0% to <0.6%	Ref	Ref
q2: 0.6% to <1.2%	8.5 (0.4, 16.6)	0.040**
q3: 1.2% to <2.0%	4.6 (−4.8, 14.1)	0.337
q4: 2.0% to ≤13.6%	10.4 (−0.5, 21.2)	0.061*
Percent of patients with other substance‐related disorder diagnosis, %		
q1: 0% to <0.8%	Ref	Ref
q2: 0.8% to <1.7%	−1.7 (−9.9, 6.6)	0.695
q3: 1.7% to <3.3%	2.8 (−7.0 12.6)	0.575
q4: 3.3% to ≤28.9%	4.8 (−6.7, 16.3)	0.412
FQHC size, *N*		
q1: <6041 patients	Ref	Ref
q2: 6041 to <12,474 patients	7.4 (−0.4, 15.2)	0.063*
q3: 12,474 to <24,843 patients	6.4 (−2.1, 15.0)	0.141
q4: 24,842 to ≤131,237 patients	10.3 (0.7, 20.0)	0.035**
Urban service area (ref = rural)	1.3 (−6.1, 8.8)	0.723
Total revenue ($)/1000 patients served	−0.8 (−3.8, 2.3)	0.608
FQHC has Medicaid‐managed care contract	−7.3 (−14.2, −0.3)	0.040**
Medicaid expansion state as of 2020	0.8 (−7.0, 8.5)	0.844

*Note*: This table reports percentage point differences for each quartile (where q1 is quartile 1, etc.) from multivariable linear probability models. FQHC is federally‐qualified health center. SOGI is sexual orientation/gender identity. EHR is an electronic health record. FQHCs located in United States territories have been excluded from the entire analysis (*N* = 1342).

* *p* < 0.10.

** *p* < 0.05.

*** *p* < 0.01.

### 3.3 Barriers to screening implementation

**FIGURE 2:**
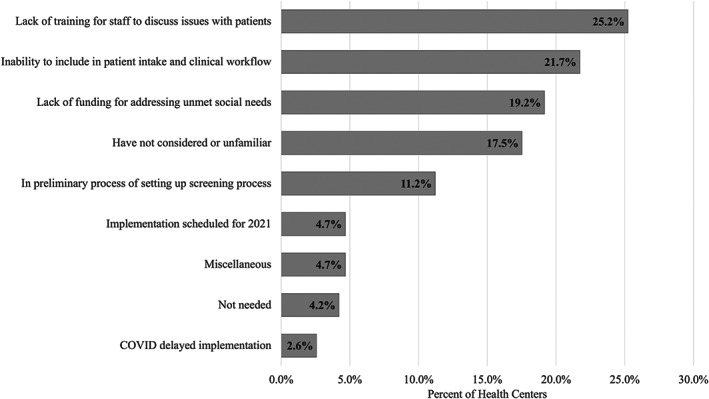
Barriers to using a standardized screening tool among federally qualified health centers not collecting social risk data (*N* = 428). Percentages do not add to 100% because federally qualified health centers were able to select multiple response options.

Of FQHCs that did not screen for social risk factors (*n* = 428), 25.2% cited that their staff lacked the training needed to discuss related issues with patients (Figure 2). Other common barriers to screening included not being able to incorporate screening into the patient intake and clinical workflow (21.7%) and insufficient funding to address unmet social needs (19.2%). Nearly one‐fifth (17.2%) of these FQHCs reported that they had not considered standardized screening assessments or were unfamiliar with them. Some FQHCs reported being in the process of setting up a screening process (11.2%) or scheduling implementation for screening in 2021 (4.7%), with some noting that the pandemic had delayed implementation (2.6%). In supplemental analyses that examined barriers among screening FQHCs that were using non‐standardized tools, 23.6% reported that incorporating standardized tools into the patient intake or clinical workflow was a barrier to using these types of tools (Figure S1).

When exploring the characteristics of non‐screening FQHC by the top four reported barriers, the distribution of FQHC characteristics was similar across barrier types, with a few exceptions (Table 3). FQHCs that reported a lack of training for staff (*N* = 108) or an inability to include screening in the patient intake (*N* = 93) had smaller average revenue per patient amounts compared to FQHCs that did not report this barrier ($690 and $666, respectively). FQHCs that indicated a lack of funding to address unmet social needs (*N* = 82) were smaller in size (13,992 unique patients), reported fewer total revenues ($5,701,980), or were located in Medicaid expansion states (75.0%). FQHCs that had not considered or were unfamiliar with standardized screener tools (*N* = 75) also tended to be smaller in size (13,626 unique patients) or be located in rural areas (56.0%) compared to FQHCs that did not report this barrier.

**TABLE 3:** Characteristics among non‐screening federally qualified health centers by top barriers to utilizing a standardized social risk assessment (2020) (*N* = 428).

	Screening barrier			
Characteristic	Lack of training for staff to discuss these issues with patients *N* = 108	Inability to include with patient intake and clinical workflow *N* = 93	Lack of funding for addressing these unmet social needs of patients *N* = 82	Have not considered/unfamiliar with assessments *N* = 75
Age, %				
Pediatric (0–17 years)	21.9 (14.5)	21.6 (13.2)	19.2 (14.0)	19.9 (13.1)
Adult (18–64 years)	64.2 (12.5)	63.4 (10.5)	65.2 (12.9)	64.5 (12.3)
Older Adult (65+ years)	11.3 (7.1)	12.3 (6.6)	12.3 (7.5)	11.5 (7.2)
Gender, %				
Female	55.9 (7.5)	56.8 (5.1)	56.0 (7.2)	56.0 (7.1)
Race/Ethnicity, %				
American Indian or Alaska Native, non‐Hispanic	2.1 (8.2)	1.8 (7.9)	3.7 (13.2)	4.9 (15.9)
Asian, non‐Hispanic	4.2 (10.0)	4.0 (10.7)	4.9 (12.5)	2.0 (4.4)
Black, non‐Hispanic	17.5 (22.8)	16.2 (19.1)	17.9 (23.7)	16.5 (22.1)
Hispanic	29.0 (27.0)	23.5 (25.0)	26.9 (26.2)	28.4 (32.8)
Other, non‐Hispanic	1.3 (1.4)	1.3 (1.7)	1.3 (1.5)	0.9 (1.3)
White, non‐Hispanic	35.0 (28.7)	44.6 (30.1)	38.0 (29.8)	38.3 (30.6)
Coverage, %				
Medicaid	39.8 (21.7)	40.8 (19.8)	38.1 (19.1)	38.8 (20.0)
Medicare	10.9 (8.3)	12.6 (8.4)	11.7 (8.6)	11.4 (8.1)
Public (e.g., state government programs)	1.0 (3.6)	0.3 (0.9)	0.4 (1.2)	1.2 (4.1)
Private	20.4 (14.0)	23.5 (14.3)	24.2 (16.0)	22.0 (14.8)
Uninsured	28.0 (20.8)	22.8 (17.4)	25.6 (18.8)	26.7 (21.1)
Patient characteristics, %				
Sexual orientation/gender identity minority	10.0 (15.1)	8.1 (11.9)	8.6 (13.9)	6.6 (9.5)
Experiencing homelessness	7.4 (19.0)	5.8 (16.3)	6.2 (15.7)	5.8 (16.1)
Best served in non‐english language	21.4 (23.7)	19.4 (24.7)	17.4 (20.8)	20.9 (29.0)
Veteran	1.7 (2.1)	1.8 (1.8)	1.6 (1.8)	1.6 (1.9)
Diabetes diagnosis	10.3 (4.9)	9.8 (3.7)	9.9 (4.6)	11.2 (5.7)
Hypertension diagnosis	19.4 (9.0)	20.6 (8.6)	19.3 (8.8)	22.3 (11.7)
Anxiety disorder diagnosis	9.2 (6.5)	10.8 (6.8)	9.3 (6.6)	10.6 (6.6)
Depression diagnosis	9.0 (6.8)	10.5 (7.2)	9.1 (6.4)	9.9 (7.3)
Alcohol‐related diagnosis	1.6 (2.8)	1.7 (2.6)	1.7 (2.9)	2.8 (8.8)
Other substance‐related disorder diagnosis	2.6 (5.8)	2.6 (4.0)	2.5 (4.8)	3.6 (9.4)
FQHC‐level characteristics				
Section 330(e) Community Health Center Grant Funding, %	92.6 (26.3)	95.7 (20.4)	96.3 (18.9)	97.3 (16.2)
Section 330(h) Health Care for the Homeless Grant Funding, %	22.2 (41.8)	17.2 (38.0)	22.0 (41.7)	18.7 (39.2)
Section 330(g) Migrant Health Center Grant Funding, %	13.0 (33.8)	14.0 (34.9)	8.5 (28.1)	10.7 (31.1)
Section 330(i) Public Housing Primary Care Grant Funding, %	0.9 (9.6)	5.4 (22.7)	2.4 (15.5)	5.3 (22.6)
Rural, %	41.7 (49.5)	47.31 (50.2)	46.3 (50.2)	56.0 (50.0)
Patient count, *n*	15,255 (19,064)	17,830 (25,926)	13,992 (19,943)	13,626 (14,707)
Total non‐patient revenue, $	6,418,546 (5,270,431)	6,749,288 (6,893,505)	5,701,980 (5,210,952)	6,648,134 (6,137,270)
Revenue per patient, $	690 (6.9)	666 (9.2)	708 (7.43)	789 (9.2)
EHR Use, %	99.1 (9.6)	100 (0)	98.8 (11.0)	98.7 (11.6)
Medicaid‐managed care contract, %	30.6 (46.3)	28.0 (45.1)	26.8 (44.6)	26.7 (44.5)
State‐level characteristics, %				
Medicaid expansion as of 2020	63.7 (48.3)	68.9 (46.6)	75.0 (43.6)	62.7 (48.7)

*Note*: This table reports proportional means and standard deviations except where otherwise noted. EHR is an electronic health record. Total Non‐Patient Revenue includes income‐related non‐patient receipts (e.g., grants and contracts). Medicaid expansion percentages exclude Federally qualified health centers located in United States territories (*N* = 1342).

## 4 DISCUSSION

This analysis of national FQHC data found that while most FQHCs were collecting some type of social risk data from patients in 2020, there were important FQHC characteristics that were associated with screening likelihood. Having a higher proportion of patients best served in a language other than English or being larger in size were characteristics significantly associated with an increase in screening likelihood, after adjusting for other characteristics. Having a higher concentration of medically uninsured patients or participating in Medicaid‐managed care contracts was significantly associated with a decrease in screening likelihood. While the PRAPARE tool was the most common standardized screening tool used among FQHCs, nearly half of all screening FQHCs reported using a different type of standardized assessment (e.g., state initiative‐specific tools) or a non‐standardized screener to collect this data. Among non‐screening FQHCs, the most common barriers to utilizing a standardized social risk screener included a lack of staff training, workflow integration capacity, funding, and familiarity with available tools. Lack of training and workflow integration capacity were top barriers among FQHCs with lower per‐patient revenue amounts, whereas lack of funding was a top barrier among smaller FQHCs and those with less total revenues and that were located in Medicaid expansion states. Lack of familiarity with standardized tools was a top barrier among smaller and rural FQHCs.

Despite the onset of the COVID‐19 pandemic in 2020, the rate of social risk screening adoption among FQHCs did not appear to be impacted significantly: an earlier study found that 71% of US FQHCs screened for social risks in 2019,^13^ compared to 69% found in our 2020 study. Although 1 in 3 US FQHCs still did not screen for social risk factors in 2020, FQHC social risk screening rates identified by our study appear much higher than screening adoption rates in other primary care settings, as reported in prior work.^25^, ^26^ Thus, our analysis of UDS data suggests that FQHCs likely remain leaders in social risk data collection despite the impact of the pandemic.

Additionally, our present findings that larger FQHCs had a higher likelihood of screening while those involved in a Medicaid‐managed care contract had a lower likelihood of screening align with findings from prior work.^13^ However, while the association between FQHC characteristics and their collection of social risk data has been studied previously, our study is the first‐known national summary of barriers to collecting standardized patient social risk data at FQHCs and provides an important window into FQHCs' experiences following the onset of the pandemic. Other work that has examined barriers to social risk screening among FQHCs has been regional, which captures the nuances of screening facilitators and barriers within specific communities; however, our study leverages a national census of FQHCs to provide a comprehensive overview of the current state of screening.^27^, ^28^, ^29^ Quantifying barriers at a national level can also help identify potential federal policy levers that mitigate barriers and facilitate the implementation of standardized screening practices across US communities.

Study results suggest that improved rates of patient health insurance coverage may strengthen FQHCs' abilities to address patients' unmet health and social needs. FQHCs deliver comprehensive care regardless of a patient's insurance status or ability to pay, which may be especially important for uninsured patients who are more likely to have unmet social needs.^24^ However, FQHCs with high concentrations of uninsured patients may have to utilize a larger share of their Health Center Program grants to pay for services that these patients receive at the FQHC because these patients do not have insurance that can be billed. Less revenue, in turn, may limit an FQHC's financial flexibility to enact initiatives related to social risk screening or engage in cross‐sector collaborations. Our findings also suggest that larger FQHCs have a higher likelihood of screening; it is likely that larger FQHCs have more total revenues and thus may be better positioned to implement interventions that require upfront investments. To counteract this resource challenge, states have the option to move toward alternative payment methodologies that can sometimes better reflect the level and cost of care that FQHCs provide. The financial flexibility of these payment models may help facilitate initiatives such as social risk screening and community collaboration to meet identified social needs; however, many FQHCs remain under the PPS.^30^, ^31^ State decisions to expand Medicaid may also benefit FQHCs with the largest shares of uninsured individuals and provide FQHCs with additional revenue to invest in activities such as social risk screening or community partnership engagement.^32^, ^33^

Interestingly, participation in a Medicaid‐managed care contract—where FQHCs contract with a privately‐administered health plan that provides Medicaid enrollees with comprehensive services in exchange for a capitated monthly payment from the state—was associated with a lower likelihood of screening. Of note, this relationship was observed in our adjusted model, while our unadjusted descriptive statistics did not find any difference in managed care participation between FQHCs that did versus did not adopt screening. Nonetheless, this finding highlights that, while managed care contracts are tools for promoting social risk screening among FQHCs, they alone may not be enough to ensure screening. Rather, managed care contracts, and other alternative payment models available to FQHCs, may need to specifically incentivize social risk screening among contracted entities and provide financing for interdisciplinary care teams that are trained to support social risk screening initiatives and engagement in cross‐sector efforts.^34^, ^35^

Study results also suggest that having a greater proportion of patients best served in a language other than English was associated with a higher likelihood of social risk screening, which underscores the important role FQHCs play as central safety‐net providers. In immigrant communities, FQHCs likely served as a critical source of comprehensive care early in the pandemic. FQHCs historically have had deep ties to their communities and have provided holistic care, such as culturally and linguistically appropriate care, free or discounted care, and connection to social services. FQHCs serving large immigrant populations may be well suited to collecting social risk data from their patients because they may already have internal resources and external partnerships specific to immigrant populations.^36^ They may also be seen as trusted providers during a time when there is increasing anti‐immigrant rhetoric and policies stemming from the Trump administration, which has led to disenrollment by immigrant families from essential public programs that address individuals' social and medical needs, such as the Supplemental Nutrition Assistance Program and Medicaid, out of fear that their immigration status would be adversely impacted if they remained in these programs.^37^, ^38^, ^39^ Higher rates of social risk screening among FQHCs with large immigrant patient populations in 2020 may suggest that these FQHCs have built strong relationships with other community‐based organizations that could support their patients, such as those who may have disenrolled from public programs, and thus may have been more motivated to screen their patients for social risks in response to the limited governmental support available to this population. However, a recent national study that explored racial, ethnic, and language differences in social risk screening among FQHCs found that the likelihood of social risk screening varied by patient race/ethnicity within the same language preference group, pointing to existing inequities in social risk screening practices that are important to examine further.^40^

Our study found that FQHCs use a variety of tools to screen for social risk factors, which may indicate FQHC responsiveness to patient needs but may also be a challenge for cross‐sector collaboratives seeking to engage FQHCs in standardized data collection and sharing efforts. While nearly half of FQHCs are utilizing the PRAPARE tool, a standardized social risk data collection tool designed for health center use, just as many FQHCs are utilizing other standardized assessments or non‐standardized tools. These findings are consistent with patterns observed in 2019.^13^ The use of standardized data collection tools may facilitate important elements of cross‐sector collaboration, such as data sharing and integration across partners,^41^ but standardization may not be effective or appropriate for every community or may be burdensome to FQHC staff. For example, the PRAPARE tool is designed for and commonly used by FQHCs for standardized social risk data collection. PRAPARE includes a comprehensive set of measures that are mapped with standardized codification sets (such as ICD‐10, LOINC, and SNOMED codes), which could facilitate the integration of data collection in the clinic setting.^42^ However, research has also documented limitations to the PRAPARE tool that may impact its acceptability, such as the length of the full version of the tool and challenges with standardized and accurate data entry in the EHR.^43^, ^44^ These types of limitations are not unique to any single standardized social risk screening tool.^45^, ^46^ As a result, some FQHCs that decide to implement social risk screening may instead adapt or create tools that better fit their needs. In particular, prior work has found that FQHCs will tailor screening tools to better reflect the issues that are most salient to their patients or to address certain organizational barriers, like limited staff availability or established workflows that may not support screening activities.^27^, ^47^ This study found similar trends emerge in an analysis of barriers to utilizing standardized screening tools among FQHCs that reported collecting social risk data with a non‐standardized tool.

Among FQHCs that were not screening for social risk factors, the top three barriers that FQHCs identified to implementing standardized social risk screening tools were all rooted in resource limitations, which suggests that creating policies designed to improve FQHCs' access to additional funding and training opportunities may be a key way to support their data collection efforts. The barriers that this study identified also reinforce the results of other studies that have examined FQHC barriers toward engaging in screening efforts, and even cross‐sector data sharing and integration efforts.^48^, ^49^ Current visit‐based FQHC payment models limit FQHCs' ability to support patients with unmet social needs through direct intervention, as these services are not covered by the visit rate. Prior work has also identified the importance of enabling staff, such as community health workers and patient navigators, to championing health care screening training and implementation.^27^, ^50^ However, these positions are not traditionally covered by the current payment model.^51^ Consequently, FQHCs' implementation of social risk screening tools or engagement in cross‐sector efforts may be limited if these activities require significant contribution of staff time and resources outside of care delivery that is explicitly covered under the current payment system.^30^ Reducing barriers to the adoption of social risk screening will require concurrent strategies, such as providing technical assistance and training to help FQHCs integrate screening into existing workflows, giving FQHCs additional grants, and encouraging more flexible value‐based payment models.^52^ Importantly, there is no clear evidence about the advantages and disadvantages of implementing a standardized versus non‐standardized social risk screener, particularly in terms of patient outcomes. It is possible, for example, that standardized screening may benefit potential organizations partnering with multiple FQHCs. However, FQHCs must consider the needs of their patient populations, workforce, and workflow and could therefore benefit from more tailored, non‐standardized screening initiatives.

Our study has limitations. First, the quality of UDS reporting may have been negatively affected by shifts in FQHCs' focus toward COVID‐19 testing and care in 2020. However, social risk data collection efforts appeared stable compared to the prior year.^13^ Second, though standardized, the UDS data are self‐reported and therefore are subject to reporting errors. While HRSA provides reporting guidance to FQHCs, the individuals responsible for submitting data can vary widely in role and capacities (e.g., FQHCs may rely on internal and/or external staff to support reporting), organizations may differently interpret what constitutes standardized social risk screening,^53^ and workforce‐related challenges may impact reporting accuracy (e.g., staff turnover across years), although the direction of potential bias is unlikely to be systematic. Finally, our findings are descriptive in nature, and the format of the UDS reporting is largely close‐ended and limits our ability to capture a fuller scope of potential barriers FQHCs may be facing to screening for social risk factors.

## 5 CONCLUSION

This study highlights key insights about FQHCs' social risk screening activities, and barriers to screening, during the first year of COVID‐19—a period of heightened social risk within low‐income communities served by FQHCs. We find that a majority of FQHCs report collecting social risk data in 2020, but that the likelihood of engaging in these efforts may vary based on certain FQHC characteristics. In particular, FQHCs with higher proportions of uninsured patients or that are smaller in size are less likely to screen, and many FQHCs face barriers to implementing standardized social risk screening tools in their practices. These are important considerations for any cross‐sector collaborations seeking to engage FQHCs in efforts aimed at advancing equity and addressing the disparities exacerbated by the COVID‐19 pandemic. As policy leaders consider ways to better address unmet social needs within marginalized populations, it will be important for them to help FQHCs overcome existing barriers to screening, including through policies that expand the financial resources and support necessary to implement social risk screening tools.

## CONFLICT OF INTEREST STATEMENT

There are no other conflicts to disclose.

## Supporting information


**Data S1.** Supporting Information.
