# Supplementary material for: Use of and barriers to adopting standardized social risk screening tools in federally qualified health centers during the first year of the COVID‐19 pandemic
Source: Health Serv Res. 2023 Sep 16;59(Suppl 1):e14232. doi: 10.1111/1475-6773.14232 (PMC10796290; doi:10.1111/1475-6773.14232)
Supplement: Supplementary file 1 — Data S1. Supporting Information. [file HESR-59-e14232-s001.docx]

**Supplemental Appendix Tables**

Supplemental Table 1. Federally Qualified Health Center Characteristics by Disaggregated Social Risk Factor Screening Status – Survey-Provided Response Options (2020)

| **Characteristic** | **Don’t Screen & Not Planning**  N=85 (6.2%) | **Don’t Screen but Planning**  N=343 (24.9%) | **Currently Screen**  N=947 (68.9%) | **P-Value of difference** |
| --- | --- | --- | --- | --- |
| **Age, %** | | | | |
| Pediatric (0-17 years) | 20.5 (15.7) | 21.4 (13.5) | 22.6 (12.6) | .143 |
| Adult (18-64 years) | 63.3 (12.7) | 64.3 (11.5) | 64.1 (11.2) | .766 |
| Older Adult (65+ years) | 11.5 (7.9) | 11.7 (7.0) | 11.2 (6.5) | .519 |
| **Gender, %** | | | | |
| Female | 55.2 (7.4) | 56.8 (6.2) | 56.6 (6.0) | .112 |
| **Race/Ethnicity, %** | | | | |
| American Indian or Alaska Native, non-Hispanic | 5.9 (17.3) | 2.8 (11.9) | 1.3 (6.1) | <.001*** |
| Asian, non-Hispanic | 1.8 (4.8) | 2.9 (8.7) | 3.3 (9.4) | .303 |
| Black, non-Hispanic | 16.4 (22.3) | 20.0 (24.2) | 17.8 (21.9) | .223 |
| Hispanic | 23.1 (26.2) | 26.3 (27.5) | 27.9 (26.8) | .218 |
| Other, non-Hispanic | 1.1 (1.6) | 1.2 (1.7) | 1.3 (2.4) | .299 |
| White, non-Hispanic | 42.3 (30.0) | 39.4 (29.8) | 41.5 (30.1) | .493 |
| **Insurance Coverage, %** | | | | |
| Medicaid | 37.2 (20.9) | 39.7 (19.6) | 43.2 (17.3) | <.001*** |
| Medicare | 11.5 (8.5) | 11.8 (7.9) | 11.8 (7.2) | .920 |
| Other Public (e.g., state government programs) | 1.0 (3.4) | 0.6 (2.4) | 0.7 (2.1) | .378 |
| Private | 23.1 (14.4) | 21.4 (13.3) | 21.9 (12.8) | .535 |
| Uninsured | 27.1 (21.4) | 26.5 (19.6) | 22.4 (16.3) | <.001*** |
| **Other Patient Characteristics, %** | | | | |
| Sexual Orientation/Gender Identity Minority | 5.9 (8.0) | 8.4 (13.2) | 8.9 (13.5) | .128 |
| Experiencing Homelessness | 5.9 (18.0) | 7.0 (18.1) | 6.8 (16.6) | .870 |
| Best Served in non-English Language | 16.9 (23.6) | 18.4 (23.1) | 20.3 (22.4) | .230 |
| Veteran | 1.7 (2.0) | 1.7 (1.9) | 1.8 (2.3) | .898 |
| Diabetes Diagnosis | 9.8 (5.0) | 10.2 (4.8) | 9.8 (3.9) | .351 |
| Hypertension Diagnosis | 19.5 (10.5) | 20.3 (9.6) | 19.2 (8.3) | 0.153 |
| Anxiety Diagnosis | 9.7 (7.0) | 9.2 (6.3) | 11.2 (6.6) | .003*** |
| Depression Diagnosis | 9.6 (7.5) | 9.7 (6.8) | 10.9 (7.1) | .013* |
| Alcohol Use Diagnosis | 1.8 (3.1) | 1.7 (4.4) | 1.8 (2.4) | .911 |
| Other Substance Use Disorder Diagnosis | 2.6 (4.3) | 2.8 (5.9) | 3.1 (4.6) | .339 |
| **FQHC-level Characteristics** | | | | |
| Section 330(e) Community Health Center Grant Funding, % | 94.1 (23.7) | 95.0 (21.8) | 94.8 (22.2) | .942 |
| Section 330(h) Health Care for the Homeless Grant Funding, % | 14.1 (35.0) | 21.0 (40.8) | 22.7 (41.9) | .171 |
| Section 330(g) Migrant Health Center Grant Funding, % | 10.6 (31.0) | 13.4 (34.1) | 12.7 (33.3) | .780 |
| Section 330(i) Public Housing Primary Care Grant Funding, % | 4.7 (21.3) | 7.6 (26.5) | 8.1 (27.4) | .522 |
| Rural, % | 55.3 (50.0) | 45.2 (49.8) | 39.7 (49.0) | .008*** |
| Unique Patient Count, n | 12,862 (15,016) | 16,905 (19,924) | 22,914 (28,873) | <.001*** |
| Total Non-Patient Revenue, $ | 6,240,412 (6,209,897) | 7,939,073 (10,600,000) | 9,579,401 (15,300,000) | .030** |
| Revenue per patient, $ | 984 (13.3) | 686 (7.9) | 681 (18.7) | .259 |
| EHR Use, % | 99.8 (10.9) | 99.4 (7.6) | 99.7 (5.6) | .460 |
| Medicaid Managed Care Contract, % | 18.8 (39.3) | 25.1 (43.4) | 24.2 (42.8) | .479 |
| **State-level Characteristics, %** | | | | |
| Medicaid Expansion as of 2020 | 70.4 (46.0) | 64.6 (47.9) | 74.0 (43.9) | .004*** |

Note: ***p<.01, **p<.05, *p<.10. This table reports proportional means and standard deviations except where otherwise noted. Wilcoxon-Mann-Whitney tests were used to assess statistical differences. EHR is electronic health record. Total Non-Patient Revenue includes income-related non-patient receipts (e.g., grants and contracts). Medicaid expansion percentages exclude federally qualified health centers located in United States territories (N = 1,342).

Supplemental Table 2. Unadjusted and Adjusted Differences in the Probability of Social Risk Screening – Contiguous United States Federally Qualified Health Centers

|  | **Unadjusted** | | **Adjusted** | |
| --- | --- | --- | --- | --- |
| **Variables** | **Percentage Point Difference**  **(95% CI)** | **P-Value** | **Percentage Point Difference**  **(95% CI)** | **P-Value** |
| Percent patients who are pediatric (0 – 17 years), % |  |  |  |  |
| q1: 0% to <13.7% | ref | ref | ref | ref |
| q2: 13.7% to <22.4% | 6.7 (-0.3, 13.7) | .062* | 2.2 (-5.6, 9.9) | .587 |
| q3: 22.4% to <30.6% | 7.4 (0.4, 14.3) | .038** | -1.3 (-10.0, 7.3) | .763 |
| q4: 30.6% to ≤54.5% | 8.1 (1.2, 15.1) | .022** | 1.4 (-8.5, 11.4) | .780 |
| Percent patients who are older adults (65+ years), % |  |  |  |  |
| q1: 0% to 6.7% | Ref | ref | ref | ref |
| q2: 6.7% to <9.5% | 9.1 (2.1, 16.1) | .010* | 2.8 (-5.2, 10.8) | .486 |
| q3: 9.5% to <14.5% | 1.3 (-5.7, 8.3) | .716 | -5.6 (-15.0, 3.9) | .248 |
| q4: 14.5% to ≤32.6% | -1.3 (-8.3, 5.7) | .714 | -0.7 (-13.2, 11.9) | .919 |
| Percent patients who are American Indian/Alaska Native, non-Hispanic, % |  |  |  |  |
| q1: 0% | ref | ref | ref | ref |
| q2: >0% to <0.2% | 14.7 (7.5, 22.0) | <.001*** | 7.7 (-1.1, 16.4) | .086* |
| q3: 0.2% to <0.6% | 11.6 (5.0, 18.2) | .001*** | 3.7 (-4.0, 11.5) | .343 |
| q4: 0.6% to ≤52.6% | 6.1 (-0.6, 12.7) | .073* | 1.1 (-6.8, 9.1) | .782 |
| Percent patients who are Asian, non-Hispanic, % |  |  |  |  |
| q1: 0% to <0.3% | ref | ref | ref | ref |
| q2: 0.3% to <0.8% | 12.3 (5.3, 19.3) | .001*** | 4.3 (-3.4, 12.0) | .268 |
| q3: 0.8% to <2.2% | 9.6 (2.6, 16.7) | .007*** | 0.6 (-9.2, 8.1) | .901 |
| q4: 2.2% to ≤55.4% | 11.7 (4.6, 18.7) | .001*** | 0.7 (-8.6, 10.0) | .884 |
| Percent patients who are Black, non-Hispanic, % |  |  |  |  |
| q1: 0% to <1.6% | ref | ref | ref | ref |
| q2: 1.6% to <8.3% | 8.7 (1.6, 15.8) | .016** | 5.5 (-2.4, 13.5) | .171 |
| q3: 8.3% to <30.1% | 6.1 (-1.0, 13.1) | .092* | 2.1 (-6.9, 11.1) | .647 |
| q4: 30.1% to ≤88.0% | 2.0 (-5.1, 9.1) | .579 | 4.5 (-5.4, 14.4) | .373 |
| Percent patients who are Hispanic, % |  |  |  |  |
| q1: 0% to <4.7% | ref | ref | ref | ref |
| q2: 4.7% to <16.3% | 1.1 (-5.9, 8.0) | .763 | -4.1 (-13.4, 5.3) | .397 |
| q3: 16.3% to <42.4% | 3.4 (-3.6, 10.4) | .338 | -2.6 (-14.0, 8.8) | .653 |
| q4: 42.4% to ≤91.7% | 6.0 (-1.1, 13.1) | .096* | -0.7 (-13.6, 12.2) | .916 |
| Percent patients insured by Medicaid, % |  |  |  |  |
| q1: 0% to <27.4% | ref | ref | ref | ref |
| q2: 27.4% to <41.5% | 9.0 (2.1, 15.9) | .010** | 2.5 (-5.4, 10.4) | .537 |
| q3: 41.5% to <56.0% | 14.4 (7.4, 21.3) | <.001*** | 2.4 (-7.3, 12.1) | .627 |
| q4: 56.0% to ≤79.6% | 12.0 (5.0, 19.0) | .001*** | -2.8 (-14.5, 8.9) | .642 |
| Percent patients insured by Medicare, % |  |  |  |  |
| q1: 0% to <6.3% | ref | ref | ref | ref |
| q2: 6.3% to <10.2% | 7.1 (0.1, 14.1) | .047** | 4.3 (-4.0, 11.8) | .294 |
| q3: 10.2% to <16.4% | 8.5 (1.4, 15.5) | .018** | 7.9 (-4.5, 15.4) | .128 |
| q4: 16.4% to ≤33.4% | 1.6 (-5.4, 8.6) | .649 | 2.1 (-14.0, 11.4) | .753 |
| Percent patients who are uninsured, % |  |  |  |  |
| q1: 0% to <11.1% | ref | ref | ref | ref |
| q2: 11.1% to <19.4% | -0.2 (-7.2, 6.8) | .963 | -4.7 (-12.2, 2.7) | .211 |
| q3: 19.4% to <31.5% | -4.2 (-11.2, 2.8) | .237 | -7.7 (-16.2, 0.8) | .075* |
| q4: 31.5% to ≤81.4% | -10.8 (-17.8, -3.7) | .003*** | -14.2 (-25.5, -0.3) | .013** |
| Percent patients who identified as SOGI minority , % |  |  |  |  |
| q1: 0% to <1.7% | ref | ref | ref | ref |
| q2: 1.7% to <3.7% | 9.6 (2.5, 16.6) | .008*** | 6.3 (-1.1, 13.6) | .093* |
| q3: 3.7% to <10.0% | 8.8 (1.7, 15.8) | .015** | 3.6 (-4.0, 11.2) | .352 |
| q4: 10.0% to ≤68.5% | 8.7 (1.7, 15.8) | .016** | 5.8 (-1.6, 13.2) | .127 |
| Percent patients who are experiencing homelessness, % |  |  |  |  |
| q1: 0% to <0.3% | ref | ref | ref | ref |
| q2: 0.3% to <1.4% | 10.1 (4.0, 18.0) | .002*** | 4.8 (-2.5, 12.1) | .200 |
| q3: 1.4% to <5.0% | 10.5 (3.5, 17.5) | .003*** | 5.6 (-2.1, 13.2) | .152 |
| q4: 5.0% to ≤100% | 10.3 (3.3, 17.3) | .004*** | 5.0 (-3.1, 13.1) | .228 |
| Percent patients best served in a language other than English, % |  |  |  |  |
| q1: 0% to <2.1% | ref | ref | ref | ref |
| q2: 2.1% to <10.4% | 4.8 (-2.1, 11.7) | .176 | 6.4 (-3.0, 15.7) | .181 |
| q3: 10.4% to <29.5% | 2.8 (-4.1, 9.8) | .423 | 5.1 (-6.5, 16.6) | .389 |
| q4: 29.5% to ≤79.8% | 11.0 (4.0, 18.1) | .002*** | 18.8 (6.0, 31.6) | .004*** |
| Percent of patients with a diabetes diagnosis, % |  |  |  |  |
| q1: 0% to <7.2% | ref | ref | ref | ref |
| q2: 7.2% to <9.4% | 2.6 (-4.4, 9.6) | .465 | -0.9 (-8.8, 6.9) | .814 |
| q3: 9.4% to <12.1% | 6.3 (-0.7, 13.2) | .078* | 4.1 (-5.3, 13.6) | .392 |
| q4: 12.1% to ≤22.9% | -2.1 (-9.1, 4.9) | .558 | 3.0 (-8.2, 14.1) | .602 |
| Percent patients with a hypertension diagnosis, % |  |  |  |  |
| q1: 0% to <13.3% | ref | ref | ref | ref |
| q2: 13.3% to <18.5% | 1.7 (-5.3, 8.7) | .633 | -2.0 (-10.0, 6.1) | .631 |
| q3: 18.5% to <24.4% | 1.4 (-5.6, 8.4) | .694 | -3.4 (-13.4, 6.5) | .500 |
| q4: 24.4% to ≤45.0% | -5.8 (-12.8, 1.1) | .101 | -6.6 (19.3, 6.1) | .309 |
| Percent of patients with an anxiety disorder diagnosis, % |  |  |  |  |
| q1: 0% to <6.5% | ref | ref | ref | ref |
| q2: 6.5% to <9.8% | 7.5 (0.5, 14.5) | .037** | 4.1 (-4.5, 12.6) | .356 |
| q3: 9.8% to <13.8% | 6.1 (-0.9, 13.1) | .089* | 2.4 (-8.2, 13.0) | .660 |
| q4: 13.8% to ≤35.4% | 12.2 (5.1, 19.2) | .001*** | 7.4 (-5.3, 20.1) | .254 |
| Percent of patients with a depression diagnosis, % |  |  |  |  |
| q1: 0% to <6.1% | ref | ref | ref | ref |
| q2: 6.1% to <9.2% | 5.2 (-1.9, 12.2) | .150 | -2.2 (-11.2, 6.8) | .631 |
| q3: 9.2% to <13.1% | 6.2 (-0.8, 13.2) | .083* | -1.9 (-13.1, 9.4) | .742 |
| q4: 13.1% to ≤42.8% | 10.2 (3.2, 17.3) | .004*** | -1.7 (-15.2, 11.9) | .811 |
| Percent of patients with an alcohol-related disorder diagnosis, % |  |  |  |  |
| q1: 0% to <0.6% | Ref | ref | ref | ref |
| q2: 0.6% to <1.2% | 11.3 (4.2, 18.3) | .002*** | 8.5 (0.4, 16.6) | .040** |
| q3: 1.2% to <2.0% | 10.3 (3.2, 17.3) | .004*** | 4.6 (-4.8, 14.1) | .337 |
| q4: 2.0% to ≤13.6% | 15.9 (8.9, 22.9) | <.001*** | 10.4 (-0.5, 21.2) | .061* |
| Percent of patients with other substance-related disorder diagnosis, % |  |  |  |  |
| q1: 0% to <0.8% | ref | ref | ref | ref |
| q2: 0.8% to <1.7% | 5.6 (-1.4, 12.6) | .119 | -1.7 (-9.9, 6.6) | .695 |
| q3: 1.7% to <3.3% | 11.6 (4.6, 18.6) | .001*** | 2.8 (-7.0 12.6) | .575 |
| q4: 3.3% to ≤28.9% | 14.3 (7.3, 21.3) | <.001*** | 4.8 (-6.7, 16.3) | .412 |
| FQHC size, N |  |  |  |  |
| q1: <6041 patients | ref | ref | ref | ref |
| q2: 6041 to <12,474 patients | 10.1 (3.2, 17.1) | .004*** | 7.4 (-0.4, 15.2) | .063* |
| q3: 12,474 to <24,843 patients | 11.8 (4.9, 18.7) | .001*** | 6.4 (-2.1, 15.0) | .141 |
| q4: 24,842 to ≤131,237 patients | 17.3 (10.4, 24.2) | <.001*** | 10.3 (0.7, 20.0) | .035** |
| Urban service area (ref=rural) | -5.4 (-10.4, -0.4) | .036** | 1.3 (-6.1, 8.8) | .723 |
| Total revenue ($)/1,000 patients served | -3.1 (-5.8, 0.5) | .021** | -0.8 (-3.8, 2.3) | .608 |
| FQHC has Medicaid managed care contract | 0.0 (-5.9, 5.8) | .994 | -7.3 (-14.2, -0.3) | .040** |
| Medicaid expansion state as of 2020 | 8.7 (3.3, 14.2) | .002** | 0.8 (-7.0, 8.5) | .844 |

Note: ***p<.01, **p<.05, *p<.10. This table reports percentage point differences for each quartile (where q1 is quartile 1, etc.) from multivariable linear probability models. FQHC is federally qualified health center. SOGI is sexual orientation/gender identity. EHR is electronic health record. FQHCs located in United States territories have been excluded from the entire analysis (N = 1,342).

Supplemental Table 3. Unadjusted and Adjusted Differences in the Probability of Social Risk Screening – All Federally Qualified Health Centers

|  | **Unadjusted** | | **Adjusted** | |
| --- | --- | --- | --- | --- |
| **Variables, %** | **Estimated Difference (95% CI)** | **P-Value** | **Estimated Difference (95% CI)** | **P-Value** |
| Percent patients who are pediatric (0 – 17 years) |  |  |  |  |
| q1: 0% to <13.8% | ref | ref | ref | ref |
| q2: 13.8% to <22.4% | 6.7 (-0.2, 12.6) | .058* | 2.3 (-5.4, 10.1) | .552 |
| q3: 22.4% to <30.6% | 7.0 (0.1, 13.9) | .048** | -1.5 (-10.0, 7.1) | .736 |
| q4: 30.6% to ≤54.5% | 7.2 (0.3, 14.1) | .042** | 1.0 (-8.7, 10.7) | .839 |
| Percent patients who are older adults (65+ years) |  |  |  |  |
| q1: 0% to <6.7% | ref | ref | ref | ref |
| q2: 6.7% to <9.5% | 8.4 (1.5, 15.3) | .017** | 2.5 (-5.4, 10.3) | .537 |
| q3: 9.5% to <14.6% | 1.7 (-5.2, 8.7) | .621 | -5.2 (-14.5, 4.2) | .275 |
| q4: 14.6% to ≤32.6% | -2.1 (-9.1, 4.8) | .544 | -2.2 (-14.4, 10.1) | .729 |
| Percent patients who are American Indian/Alaska Native |  |  |  |  |
| q1: 0% | ref | ref | ref | ref |
| q2: >0% to <0.2% | 14.9 (7.8, 22.0) | <.001*** | 7.5 (-1.1, 16.2) | .087* |
| q3: 0.2% to <0.5% | 11.6 (5.1, 18.1) | .001*** | 3.6 (-4.1, 11.2) | .357 |
| q4: 0.5% to ≤52.6% | 6.2 (-0.3, 12.8) | .061* | 1.2 (-6.6, 9.1) | .759 |
| Percent patients who are Asian, non-Hispanic |  |  |  |  |
| q1: 0% to <0.3% | ref | ref | ref | ref |
| q2: 0.3% to <0.8% | 11.1 (4.1, 18.0) | .002*** | 3.3 (-4.2, 10.8) | .391 |
| q3: 0.8% to <2.2% | 8.7 (1.8, 15.6) | .013** | -1.8 (-10.3, 6.7) | .676 |
| q4: 2.2% to ≤55.4% | 10.4 (3.5, 17.3) | .003*** | -1.0 (-10.1, 8.0) | .822 |
| Percent patients who are Black, non-Hispanic |  |  |  |  |
| q1: 0% to <1.4% | ref | ref | ref | ref |
| q2: 1.4% to <7.8% | 9.0 (2.1, 15.9) | .011** | 4.8 (-2.8, 12.5) | .216 |
| q3: 7.8% to <28.8% | 6.4 (-0.5, 13.3) | .070* | 1.1 (-7.6, 9.8) | .804 |
| q4: 28.8% to ≤88.0% | 1.9 (-5.0, 8.9) | .583 | 2.9 (-6.7, 12.5) | .554 |
| Percent patients who are Hispanic |  |  |  |  |
| q1: 0% to <4.7% | ref | ref | ref | ref |
| q2: 4.7% to <16.5% | 1.7 (-5.2, 8.67) | .621 | -3.38 (-12.6, 5.8) | .471 |
| q3: 16.5% to <44.0% | 4.1 (-2.9, 11.0) | .249 | -1.83 (-12.9, 9.2) | .745 |
| q4: 44.0% to ≤99.2% | 6.9 (0.0, 13.8) | .051* | 0.54 (-11.7, 12.8) | .931 |
| Percent patients insured by Medicaid |  |  |  |  |
| q1: 0% to <27.4% | Ref | ref | ref | ref |
| q2: 27.4% to <41.8% | 9.3 (2.4, 16.2) | .008*** | 2.7 (-5.1, 10.4) | .497 |
| q3: 41.8% to <56.2% | 14.5 (7.7, 21.4) | <.001*** | 2.5 (-6.9, 11.8) | .602 |
| q4: 56.2% to ≤81.6% | 12.1 (5.2, 19.0) | .001*** | -2.9 (-14.2, 8.4) | .617 |
| Percent patients insured by Medicare |  |  |  |  |
| q1: 0% to <6.2% | ref | ref | ref | ref |
| q2: 6.2% to <10.2% | 7.9 (0.9, 14.8) | .026** | 4.5 (-3.5, 12.5) | .267 |
| q3: 10.2% to <16.3% | 8.7 (1.8, 15.6) | .013** | 7.3 (-2.7, 17.3) | .153 |
| q4: 16.3% to ≤33.4% | 1.9 (-5.0, 8.9) | .583 | 2.0 (-10.9, 14.8) | .763 |
| Percent patients who are uninsured |  |  |  |  |
| q1: 0% to <10.9% | ref | ref | ref | ref |
| q2: 10.9% to <19.3% | 0.3 (-6.6, 7.2) | .934 | -4.4 (-11.7, 2.8) | .233 |
| q3: 19.3% to <31.5% | -3.5 (-10.4, 3.4) | .322 | -7.4 (-15.6, 0.8) | .077* |
| q4: 31.5% to ≤83.9% | -10.9 (-17.8, -4.0) | .002*** | -14.8 (-25.6, -3.9) | .008*** |
| Percent patients who identified as SOGI minority |  |  |  |  |
| q1: 0% to <1.6% | ref | ref | ref | ref |
| q2: 1.6% to <3.6% | 9.6 (2.7, 16.5) | .007*** | 5.7 (-1.5, 12.9) | .122 |
| q3: 3.6% to <9.7% | 9.0 (2.1, 15.9) | .011** | 3.4 (-4.1, 10.9) | .374 |
| q4: 9.7% to ≤68.5% | 9.2 (2.3, 16.1) | .009*** | 5.7 (-1.7, 13.0) | .131 |
| Percent patients who are experiencing homelessness |  |  |  |  |
| q1: 0% to <0.3% | ref | ref | ref | ref |
| q2: 0.3% to <1.3% | 11.1 (4.2, 17.9) | .002*** | 4.7 (-2.5, 11.9) | .198 |
| q3: 1.3% to <4.8% | 11.3 (4.4, 18.2) | .001*** | 6.0 (-1.6, 13.5) | .120 |
| q4: 4.8% to ≤100% | 11.2 (4.4, 18.2) | .001*** | 5.5 (-2.5, 13.6) | .178 |
| Percent patients best served in a language other than English |  |  |  |  |
| q1: 0% to <2.2% | ref | ref | ref | ref |
| q2: 2.2% to <10.9% | 4.65 (-2.3, 11.6) | .187 | 5.9 (-3.4, 15.1) | .214 |
| q3: 10.9% to <31.0% | 2.33 (-4.6, 9.2) | .509 | 3.9 (-7.3, 15.2) | .494 |
| q4: 31.0% to ≤99.8% | 10.39 (3.5, 17.3) | .003*** | 18.2 (5.8, 30.5) | .004*** |
| Percent of patients with a diabetes diagnosis |  |  |  |  |
| q1: 0% to <7.2% | ref | ref | ref | ref |
| q2: 7.2% to <9.4% | 2.9 (-4.0, 9.8) | .410 | -0.8 (-8.6, 6.9) | .835 |
| q3: 9.4% to <12.1% | 6.7 (-2.3, 13.6) | .058* | 3.9 (-5.3, 13.1) | .408 |
| q4: 12.1% to ≤22.9% | -1.6 (-8.5, 5.4) | .660 | 2.1 (-8.8, 13.0) | .700 |
| Percent of patients with a hypertension diagnosis |  |  |  |  |
| q1: 0% to <13.3% | ref | ref | ref | ref |
| q2: 13.3% to <18.5% | 1.5 (-5.5, 8.4) | .680 | -2.2 (-10.1, 5.8) | .593 |
| q3: 18.5% to <24.3% | 2.3 (-4.6, 9.3) | .510 | -2.1 (-11.9, 7.7) | .674 |
| q4: 24.3% to ≤45.0% | -5.1 (-12.0, 1.9) | .153 | -4.9 (-17.4, 7.6) | .444 |
| Percent of patients with an anxiety disorder diagnosis, % |  |  |  |  |
| q1: 0% to <6.3% | ref | ref | ref | ref |
| q2: 6.3% to <9.6% | 9.0 (2.1, 15.9) | .010** | 5.2 (-3.2, 13.6) | .224 |
| q3: 9.6% to <13.8% | 7.6 (0.7, 14.5) | .032** | 3.8 (-6.6, 14.2) | .472 |
| q4: 13.8% to ≤35.4% | 13.6 (6.7, 20.5) | <.001*** | 8.7 (-3.8, 21.2) | .171 |
| Percent of patients with a depression diagnosis, % |  |  |  |  |
| q1: 0% to <6.0% | ref | ref | ref | ref |
| q2: 6.0% to <9.1% | 6.7 (-0.2, 13.6) | .058* | -1.2 (-10.0, 7.6) | .792 |
| q3: 9.1% to <13.0% | 7.3 (0.4, 14.2) | .039** | -1.4 (-12.4, 9.6) | .800 |
| q4: 13.0% to ≤42.8% | 11.6 (4.6, 18.5) | .001*** | -1.0 (-14.2, 12.3) | .884 |
| Percent of patients with an alcohol-related disorder diagnosis, % |  |  |  |  |
| q1: 0% to <0.6% | ref | ref | ref | ref |
| q2: 0.6% to <1.1% | 11.6 (4.8, 18.5) | .001*** | 7.6 (-0.4, 15.5) | .061* |
| q3: 1.1% to <1.9% | 10.5 (3.6, 17.3) | .003** | 3.1 (-6.2, 12.4) | .511 |
| q4: 1.9% to ≤13.6% | 16.2 (9.3, 23.1) | <.001*** | 8.3 (-2.4, 19.0) | .128 |
| Percent of patients with other substance-related disorder diagnosis, % |  |  |  |  |
| q1: 0% to <0.8% | ref | ref | ref | ref |
| q2: 0.8% to <1.7% | 6.7 (-0.2, 13.6) | .057* | -0.9 (-9.0, 7.2) | .826 |
| q3: 1.7% to <3.3% | 12.8 (5.9, 19.7) | <.001*** | 4.0 (-5.6, 13.6) | .417 |
| q4: 3.3% to ≤28.9% | 15.3 (8.5, 22.2) | <.001*** | 5.5 (-5.8, 16.9) | .339 |
| FQHC size, N |  |  |  |  |
| q1: <6,084 patients | ref | ref | ref | ref |
| q2: 6,084 to <12,404 patients | 10.2 (3.3, 17.0) | .004*** | 8.4 (0.8, 16.1) | .030** |
| q3: 12,404 to <24,757 patients | 11.3 (4.5, 18.2) | .001*** | 7.1 (-1.3, 15.5) | .096* |
| q4: 24,757 to ≤131,237 patients | 18.0 (11.1, 24.8) | <.001*** | 11.8 (2.5, 21.2) | .013** |
| Urban service area (ref=rural), | -6.6 (-11.6, -1.6) | .009*** | -0.6 (-7.9, 6.7) | .874 |
| Total revenue ($)/1,000 patients served | -0.5 (-2.0, 1.0) | .500 | 0.4 (-1.2, 2.0) | .617 |
| FQHC has Medicaid managed care contract | 0.4 (-5.3, 6.1) | .888 | -7.0 (-13.9, - 0.1) | .046** |
| Medicaid expansion state as of 2020 | 9.1 (3.8, 14.5) | .001*** | 0.6 (-6.4, 7.6) | .865 |

Note: ***p<.01, **p<.05, *p<.10. This table reports percentage point differences for each quartile (where q1 is quartile 1, etc.) from multivariable linear probability models. FQHC is federally qualified health center. SOGI is sexual orientation/gender identity. EHR is electronic health record. FQHCs located in United States territories are included in the entire analysis (N = 1,375).

Supplemental Figure 1. Barriers to Using a Standardized Screening Tool Among Federally Qualified Health Centers That Screen with a Non-Standardized Tool (N=271)

Note: Percentages do not add to 100% because federally qualified health centers were able to select multiple response options. ACO is Accountable Care Organization. EHR is electronic health record.
